# Survivin Associates with VDAC2 and Bcl2-Family Proteins at the Mitochondrial Outer Membrane

**DOI:** 10.3390/ijms27135707

**Published:** 2026-06-24

**Authors:** Adesh D. Vaidya, Hilmi Arica, Hana Abdelkabir, Lolwah Alsalamah, Kirstie Coe, Sally P. Wheatley

**Affiliations:** School of Life Sciences, University of Nottingham, Nottingham NG7 2UH, UK

**Keywords:** apoptosis, Bak, Bax, Bcl-XL, Bcl2-family, mitochondria, porin, survivin, VDAC

## Abstract

Survivin is a cancer-associated inhibitor of apoptosis protein (IAP) that can suppress both extrinsic and intrinsic apoptotic pathways. IAPs typically prevent programmed cell death by binding to caspases, but whether survivin behaves as a canonical IAP or can protect cells from death by alternative means has not been fully investigated. Here, we report a novel interaction between survivin and the mitochondrial outer membrane protein, VDAC2, which we show is an indirect association potentially mediated by Bcl2-family members. This novel finding suggests survivin can suppress mitochondrial-mediated apoptosis upstream of caspases and could open a new avenue for targeting survivin in anti-cancer therapy regimes.

## 1. Introduction

**Figure 1:**
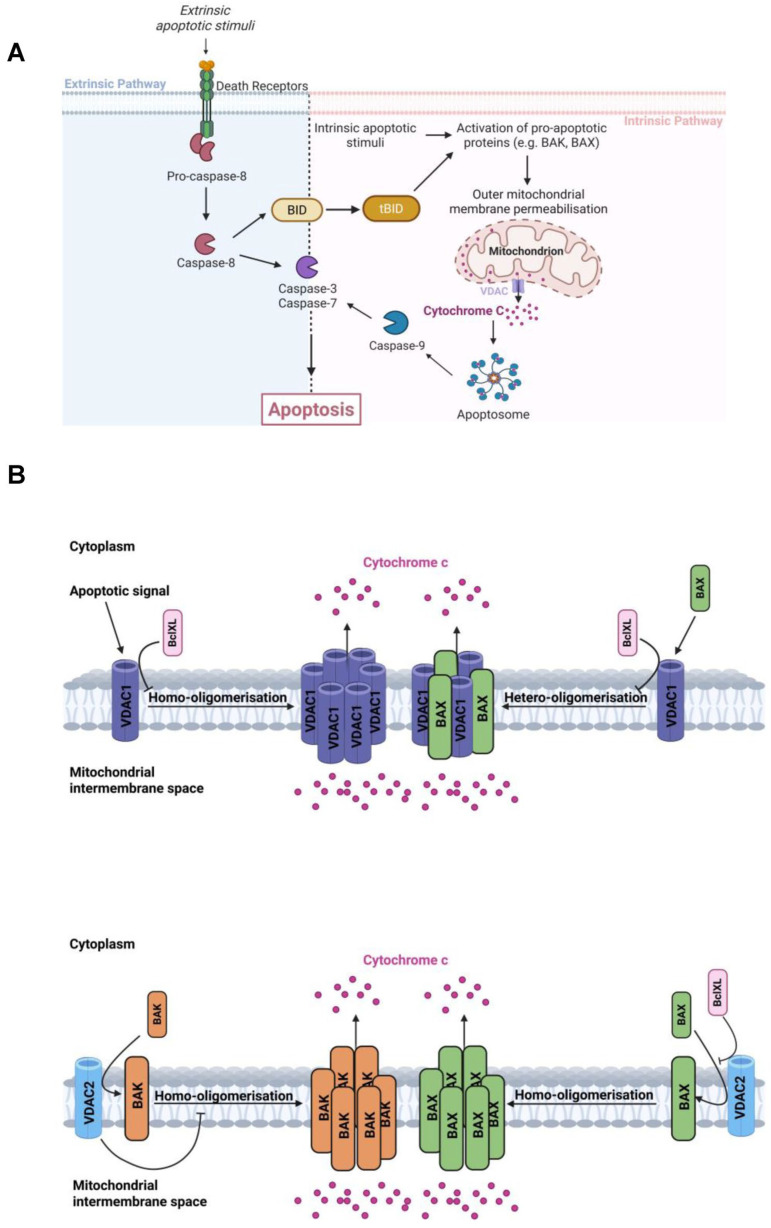
(**A**) Schematic diagrams of the 2 major apoptotic pathways. The extrinsic pathway is activated on the extracellular side of the plasma membrane (PM) by ligands that interact with death receptors. This causes the recruitment of pro-caspase 8 to the cytosolic side of the receptor, where it is activated. Caspase 8, in turn, activates the effector caspases, caspases 3 and 7, to dismantle the cell. Caspase 8 can also truncate BID, which activates it (tBID), and this leads to crosstalk with the intrinsic pathway. However, for simplicity, this will not be discussed further here. The intrinsic pathway: an external stimulus, such as IR and drug treatments, causes OMM permeabilisation via VDAC proteins, which enable cytochrome c (cytc) release into the cytoplasm. Cytc then binds to the apoptosome, which causes a conformational change that activates caspase 9. Caspase 9 then activates caspase 3 and 7, similarly to caspase 8. (**B**) Mechanism of cytc release by VDACs and Bcl2 family proteins in the intrinsic apoptotic pathway. Upper schematic shows VDAC1 (dark blue) monomers either oligomerise into hexamers to enable cytc (red) release, or recruit Bax (orange) and make heterohexamers to achieve this goal. Lower schematic illustrates the recruitment of Bak (green) or Bax (orange) by VDAC2, and how it influences their construction into multimeric complexes. Note: VDAC2 restricts Bak oligomerisation while facilitating Bax multimerisation. Meanwhile, Bcl-XL antagonises VDAC-mediated OMM recruitment of Bax (both panels). Arrows indicate movement and complex formation during these processes (Created in BioRender. H. Arica (https://BioRender.com, accessed on 18 April 2026)).

Survivin is the smallest member of the inhibitor of apoptosis protein (IAP) family, a class of proteins that inhibit caspases, the proteases responsible for dismantling cells during apoptosis (see Figure 1A). It is present at high levels in all cancers, and its expression correlates negatively with disease outcome, as reviewed in [1]. Like other IAPs, survivin can inhibit both the intrinsic and extrinsic apoptotic cascades. As outlined in Figure 1A, these programmes of cell death converge on caspases 3 and 7 but differ in that caspase 9 is the upstream activator in the intrinsic, mitochondrial-mediated pathway, whereas caspase 8 lies upstream in the extrinsic cascade (see [2]). The IAPs are thought to act predominantly in the cytoplasm, where they bind directly to caspases to prevent them from breaking down proteins. However, whether survivin can do this alone or needs to be associated with one of its IAP-family members, e.g., XIAP, remains unclear [3,4]. Importantly, while the extrinsic pathway initiates via specific receptors at the plasma membrane (PM), the intrinsic pathway is initiated by the mitochondria, which transduces signals received from external stressors, such as radiation and chemotherapeutic drugs, as well as internal damage from homeostatic processes. Upon receipt of an apoptotic stimulus, the mitochondria lose their outer mitochondrial membrane (OMM) potential and become permeabilised, a process known as “mitochondrial outer membrane permeabilization” (MOMP). MOMP enables the release of cytochrome c (cytc), a 12.3 kD protein that initiates the apoptotic cascade in the cytosol by activating the apoptosome (see Figure 1A) [5,6]. To achieve MOMP, voltage-dependent anionic channels (VDACs, also known as “porins”) in the OMM need to reorganise into higher order structures [7,8] and/or recruit proapoptotic Bcl2-family proteins [6,9].

In humans, there are 3 VDACs, and they are 70% homologous (Figure S1) [7,8]. In most cells, VDAC1 is the predominant form and is expressed at 10-fold and 100-fold higher levels than VDAC2 and VDAC3, respectively [7]. The exception is in the germ line, where VDAC3 is the most abundant [10,11]. Although VDAC monomers are functional channels that allow exchange of small molecules across the OMM, for molecules larger than 5 kD to pass, they need to assemble into oligomers or recruit other transmembrane proteins. Despite their significant homology (Figure S2), the mechanisms by which VDAC1 and 2 construct these large transition pores are surprisingly different (see Figure 1B): VDAC1 either homo-oligomerises into hexamers or recruits the pro-apoptotic protein, Bax and hetero-oligomerises with it [12,13]. In contrast, VDAC2 does not appear to self-associate, nor to interact with VDAC1; instead, the current opinion is that it recruits the pro-apoptotic Bcl2-family protein Bax to the OMM, enabling it to assemble channels, which are also homo-hexamers [6,9,14,15]. In addition to Bax, Bak is also recruited to the OMM by VDAC2, but instead of facilitating its oligomerisation as it does for Bax, VDAC2 inhibits Bak from constructing similar pores [9,16]. Thus, VDAC2 is proapoptotic in association with Bax, and anti-apoptotic in association with Bak. Once in the cytosol, cytc associates with the apoptosome, which in turn activates caspase 9, and thereafter caspases 3/7 to initiate cell suicide (see Figure 1A).

Finally, regulating the construction of these large complexes is yet another Bcl2-family protein, which presents in short (Bcl-xs) and long (Bcl-XL) forms. Bcl-xs is pro-apoptotic as it assists VDAC1 multimerisation [17]. In contrast, Bcl-XL antagonises membrane recruitment of Bax by VDACs, directing it back to the cytoplasm; thus, Bcl-XL is anti-apoptotic (see Figure 1B) [17,18].

In this paper, we test the hypothesis that survivin can influence the mitochondrial-mediated apoptotic response by interacting with VDAC proteins. We report that it binds specifically but indirectly to VDAC2 and can simultaneously associate with three Bcl2-family members.

## 2. Results

### 2.1. Survivin Delays/Suppresses Apoptosis in Response to UVC Radiation

**Figure 2:**
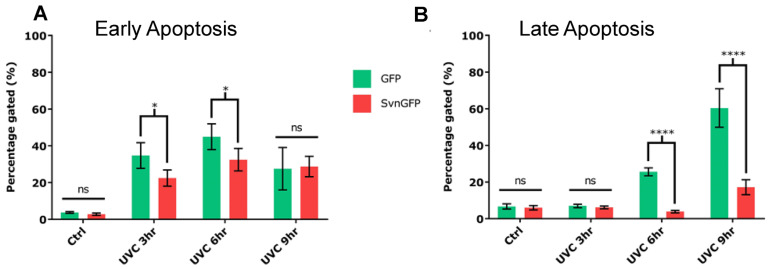
HeLa cells expressing Svn-GFP suppress UVC-induced apoptotic entry. (**A**,**B**) HeLa cells expressing GFP or survivin-GFP were treated with 180 mJ·cm^2^ of UVC (254 nm) radiation; then, at 0. 3.6 and 9 h post UVC, they were subjected to a caspase 3/7 with 7-AAD assay to identify early apoptotic and late apoptotic cells. Statistical differences for early apoptotic and late apoptotic fractions were calculated using a two-way ANOVA (ns, not significant; * *p* < 0.05, **** *p* < 0.0001). (N = 9; n = 3).

To demonstrate that overexpressing survivin can inhibit the intrinsic apoptotic pathway, we treated HeLa cells expressing GFP or survivin-GFP with 180 mJ·cm^2^ of 245 nm UVC radiation. Upon trypsin-based cell harvest at 0, 3, 6 and 9 h post-UVC, the cells were incubated with NucView^®^, a high-affinity DNA-binding dye linked to a caspase 3/7 substrate, in combination with 7-AAD, which reports the structural integrity of the PM. They were then analysed using a MUSE Cell Analyser (MERCK, Darmstadt, Germany). Based on the presence or absence of either or both caspase 3/7 and 7-AAD markers, cells were gated as either live, early apoptotic (labelled “apoptotic “ in the bottom right of Figure S1), late apoptotic/dead, or dead. A significant reduction in both early and late categories of apoptosis was seen in the UVC-treated survivin-GFP population compared to the UVC-treated GFP control, suggesting survivin can suppress or delay mitochondrial-mediated apoptosis (Figure 2 and Figure S1).

### 2.2. Survivin Interacts with VDAC

**Figure 3:**
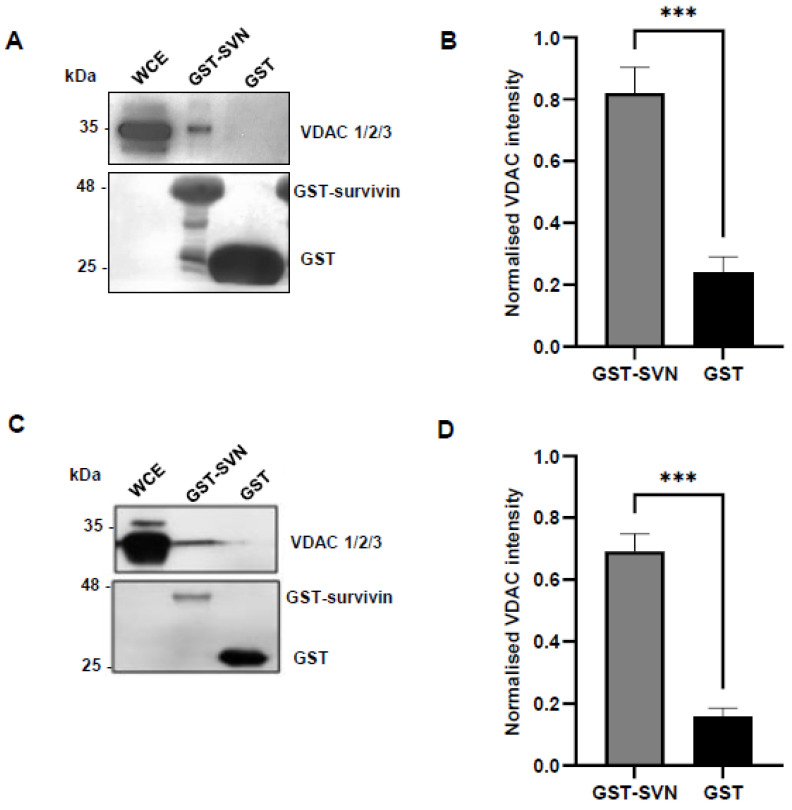
Survivin interacts with a VDAC family member. GST pull-down assays were carried out using recombinantly expressed GST and GST-survivin (SVN) in combination with WCEs made from exponentially growing (**A**) RPE or (**C**) MCF7 cells. Pan-VDAC1/2/3 signal was normalised to the corresponding GST/GST-survivin band and plotted (**B**,**D**). A significant interaction with GST-survivin, compared to the GST control, was observed in each case. N = 3. Data are mean ± SD; Student’s unpaired two-tailed *t*-test (*** *p* < 0.001).

Prompted by output data from an exploratory Mass Spectrometry (MS) screen aimed at identifying novel survivin interactors, we followed the lead hit of VDAC (see Table 1). To verify whether any VDAC family member could associate with survivin, we performed a pull-down assay using purified GST or GST-tagged survivin (GST-SVN) bound to sepharose beads, and incubated them with extracts from RPE, MCF7 (Figure 3A,B). Using a pan-VDAC 1/2/3 antibody, we observed a positive interaction with GST-SVN (43.5 kDa) in all cell extracts tested, with a band intensity significantly greater than for the negative (GST) control in each case (Figure 3B,D).

### 2.3. Survivin Interacts Simultaneously with VDAC and Bcl2 Family Members, Bcl-XL, Bak and Bax

**Figure 4:**
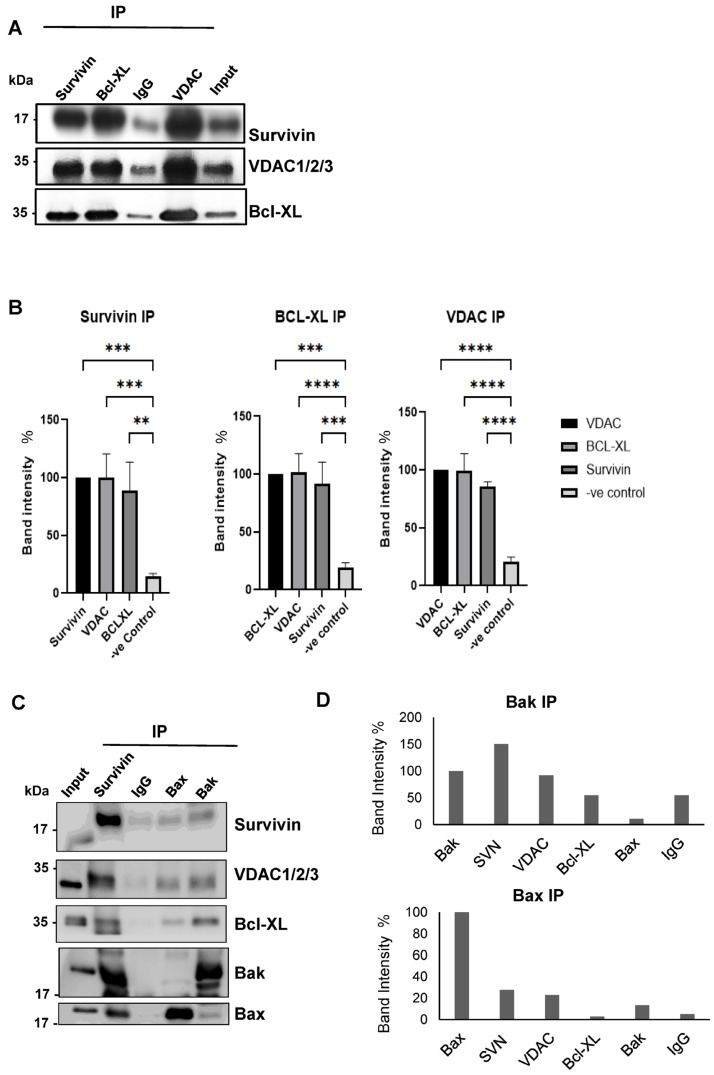
Co-Immunoprecipitations reveal simultaneous interactions between survivin, pan-VDAC1/2/3 and three Bcl2 family members. (**A**) Immunoblot analysis of reciprocal co-immunoprecipitation of survivin, pan VDAC 1/2/3 and Bcl-XL from asynchronous U2OS cell extracts. (**B**) Quantitation of (**A**), taking the band intensity of the IP’d target as 100%, and assessing the co-IP intensities in relation to it for each protein IP’d. Data are mean ± SD; statistical test, two-way ANOVA (** *p* < 0.01, *** *p* < 0.001,**** *p* < 0.0001). (**C**,**D**). The experiment was repeated with HeLa cell extracts and extended to include Bak and Bax proteins (see Figure S3A,B). Anti-survivin antibodies simultaneously co-immunoprecipitated pan VDAC 1/2/3, Bcl-XL, Bak and Bax. Bak and Bax IPs showed a similar trend but did not co-IP each other. The blots are representative of N = 2 independent repeats; therefore, no statistical test was applied.

Next, we conducted co-immunoprecipitations using antibodies to survivin, pan VDAC 1/2/3 and three Bcl2-family proteins. In our first experiment, we used Bcl-XL antibodies and asynchronous HeLa extracts. As shown in Figure 4A,B, survivin co-immunoprecipitated with antibodies to both VDAC 1/2/3 and Bcl-XL, and these associations were reciprocated in three ways, suggesting that endogenous survivin may interact simultaneously with at least one VDAC family member and Bcl-XL. When we repeated the co-IP (Figure S3A) and extended our investigation to determine whether Bak and Bax were also able to co-IP (Figure 4C), positive bands of survivin and pan VDAC1/2/3 were again witnessed. Interestingly, both Bak and Bax antibodies worked in this application, and both demonstrated clear co-IP of survivin and VDAC, but not each other (Figure 4C,D). In addition, we noted that Bax did not pull down Bcl-XL, but Bak did. Collectively, these data suggest that survivin may interact with one or more VDAC proteins when in association with Bak, Bax and/or BclXL.

### 2.4. Survivin Interacts with VDAC2 but Not with VDAC1 or VDAC3

**Figure 5:**
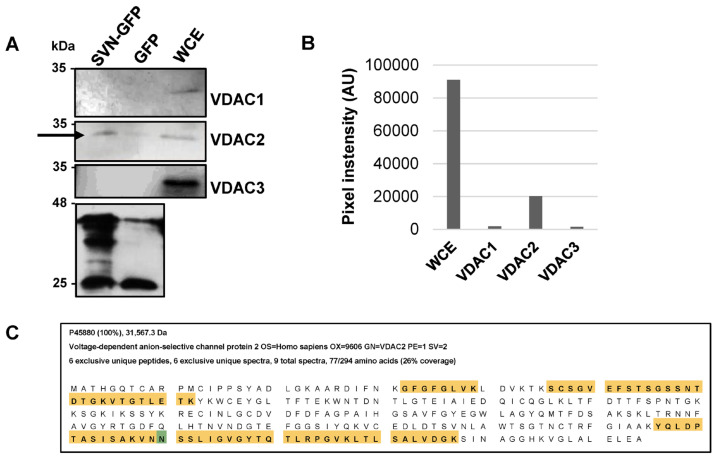
Survivin interacts with VDAC2 but not VDAC1 or 3. (**A**) GFP trap experiments were carried out using WCEs (input) from exponentially growing HeLa cells stably expressing GFP or survivin-GFP and co-immunoprecipitants probed for VDAC1, VDAC2 or VDAC3. All VDACs were expressed in the WCE, but only VDAC2 showed a positive band with survivin-GFP. N = 2 (**B**) Quantification of Survivin-GFP lanes in VDAC bands in (**A**) expressed in arbitrary units (AU). A second repeat of VDAC2 is shown in source files. Data are representative of N = 2. (**C**) Primary sequence coverage by LC-MS/MS analysis of VDAC2 protein (6 exclusive unique peptides, 77/294 amino acids, 26% coverage, yellow) following GST-survivin pull-down from HeLa cell lysate (exploratory screen). N (green) indicates deamidation.

To identify which VDAC family member(s) survivin was associating with, we carried out a GFP trap experiment, with extracts generated from exponentially growing HeLa cells stably expressing GFP or survivin-GFP. We have previously shown that survivin can support a GFP tag at its C-terminus without any detrimental effects to its normal behaviour [19]. Using VDAC1, VDAC2 or VDAC3-specific antibodies, we could detect all VDAC variants in the WCEs. However, interrogation of the GFP-trap for any co-immunoprecipitation of the VDAC family with survivin revealed a positive interaction only between VDAC2 and survivin-GFP (Figure 5A,B). This finding corroborates the initial Mass Spectrometry screen in which only VDAC2 had total spectrum count (TSC) values for GST-survivin that were above the background binding to GST control (Table 1). In fact, primary sequence coverage by LC-MS/MS analysis highlighted 6 unique and exclusive VDAC2 peptides to be present in 77/294 amino acids with 26% coverage (Figure 5C, yellow) following pull-down from asynchronous HeLa cell lysates using GST-survivin.

### 2.5. Survivin Does Not Interact Directly with VDAC2

**Figure 6:**
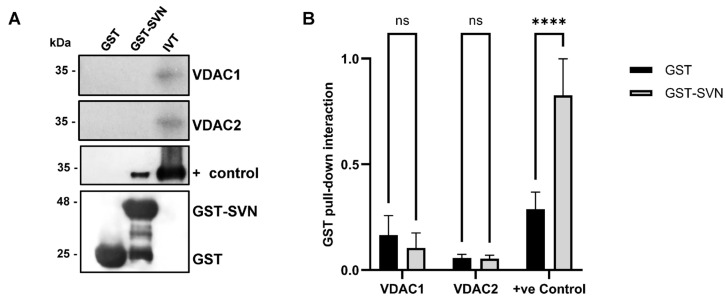
VDACs do not interact directly with survivin in vitro. GST or GST-survivin (SVN), bound to GST beads, were incubated with in vitro translated (IVT) VDAC1, VDAC2, or a positive control of similar size. Co-associating proteins were boiled off the beads and analysed by immunoblotting with anti-pan VDAC or anti-positive control (+ve control) antibodies. No interaction was observed for either VDAC, although a strong band was visible in the positive control. (**B**) Quantification of (**A**) and independent repeats (N = 3). Data are mean ± SD; Student’s unpaired two-tailed *t*-test (**** *p* < 0.0001, ns not significant).

Having established that the interaction with survivin was specific to VDAC2, next we asked whether VDAC2 could bind to survivin directly. Using pcDNA3.1 plasmids, gifted by Prof. J. Holthuis, Osnabruck [20], we in vitro translated VDAC1 and VDAC2 proteins and incubated these with GST proteins, as described for WCEs above. The results clearly demonstrated that survivin does not bind directly to VDAC2 (or VDAC1), yet a positive control of similar size did in the same conditions (Figure 6). From these experiments, we conclude that the interaction with VDAC2 is indirect and, taken together with the co-IP data, suggests that survivin interacts with VDAC2, potentially with assistance from several Bcl2 family members, including Bak, Bax and/or Bcl-XL.

## 3. Discussion

Uniquely within the IAP family, a proportion of survivin adopts mitochondrial residence in cancer cells [4]. Indeed, its ability to access these organelles in transformed, but not normal somatic cells, has led to the suggestion that mitochondrial survivin could be an “Achilles’ Heel” of cancer and targeted for therapeutic purposes [21]. Moreover, mitochondrial survivin has been reported to be more potent as an inhibitor of apoptosis than the cytosolic pool [4]. Thus, at the outset of this study, we hypothesised that in cancer cells, survivin may have an uncharacterised role further upstream in the intrinsic apoptotic pathway, rather than simply acting as a canonical IAP inhibiting caspases. The data presented herein show for the first time that survivin interacts with a single member of the porin family of OMM proteins, namely, VDAC2. This channel-forming protein regulates cytc release, which is pivotal to mitochondrial-mediated apoptotic response. However, we also discovered that this association occurs regardless of oncogenic status, as witnessed in both cancerous (U2OS, HeLa, MCF7) and non-cancerous (RPE) cells. These data suggest that the interaction occurs on the cytosolic face of the OMM rather than inside the mitochondrion per se. Although we have previously shown that survivin upregulation can increase pan-VDAC expression by interfering with PINK-parkin-mediated mitophagy [22], to our knowledge, this is the first report of survivin interacting with a porin, albeit an indirect association.

Interestingly, we also found that three Bcl2-family proteins, the pro-apoptotic proteins Bak and Bax, and the anti-apoptotic protein, Bcl-XL, can simultaneously co-IP with survivin and pan VDAC. Given the high sequence and structural homology between VDAC1 and VDAC2, the lack of VDAC1 association was surprising at first, but once we discovered Bak and Bax present in the same co-IPs, and knowing that VDAC2 assembles pores with these two Bcl2-family proteins rather than by itself or with VDAC1 (see Figure 1B), there is a clear rationale for this specificity. At this juncture, we can only speculate on several mechanisms by which survivin could inhibit apoptosis at the OMM. For example, it may act like Bcl-XL, or even in concert with Bcl-XL, to prevent VDAC2 recruitment of Bax to the OMM. Alternatively, it could prevent their oligomerisation at the OMM or bind directly to the assembled pore structure. We are currently examining which of these proteins bind to survivin directly, when these interactions form, and whether they are co-dependent or mutually exclusive liaisons within the VDAC2-survivin-Bcl2-family protein axis. Our working hypothesis is that survivin sequesters Bak and Bax in a monomeric state bound to VDAC2. While the molecular mechanism(s) involved have yet to be determined and rigorously tested, we believe that these novel data provide a turning point in our understanding of survivin as an inhibitor of the intrinsic apoptotic pathway. Clearly, this novel finding is just the beginning of an exciting new chapter in survivin biology.

## 4. Materials and Methods

### 4.1. Human Cell Culture

HeLa (cervical cancer epithelial), U2OS (osteosarcoma), RPE (retinal pigmental epithelial) and breast cancer (MCF7) cells were originally from ATCC lines. MRC5 (embryonic lung fibroblasts) were from the Genome Damage and Stability, University of Sussex, Falmer, UK. All cells were cultured at 37 °C in a 5% CO_2_ in a humidified environment in Dulbecco’s Modified Eagle’s medium (DMEM) containing 4500 mg/L of glucose, sodium pyruvate, L-glutamine and sodium carbonate (Sigma, Gillingham, UK, D6429), supplemented with 10% fetal bovine serum (Sigma, F7524) and antibiotic-antimycotic solution (Sigma, A5955). Derivative HeLa cells stably expressing GFP and survivin-GFP (first described in [19] were cultured under the selective pressure of 1 mM G418 (ACROS organics, Geel, Belgium). Mycoplasma contamination testing was conducted on all cells approximately twice per annum to ensure they were clear. The majority of experiments were carried out in U2OS and HeLa cells. RPEs and MRC5 cells were universal or cancer cell-specific.

### 4.2. Apoptosis Assay

A total of 0.4 × 10^6^ cells were seeded down into CELLSTAR^®^ 12-well plates (Greiner, Stonehouse, UK, #665180) and grown to 70–90% confluency, exposed to 180 mJ·cm^2^ of UVC (254 nm) radiation, then harvested via trypsinisation 3, 6, or 9 h post-UVC, with media retained to collect any floating cells. Cells were pelleted by centrifuging at 300× *g* for 3 min, then resuspended to a final concentration of 0.1 × 10^6^–5.0 × 10^6^ in Assay Buffer BA (Merck, Gillingham, UK, #4700-1360). Next, Muse™ Caspase 3/7 Reagent containing the caspase substrate NucView^®^ (Merck, #4700-1505) was diluted 1:8 with 1× PBS (Merck, #4700-1515), then added 1:1 with the cell suspension. Cell suspensions were then incubated for 30 min at 37 °C. A working solution of Muse™ 7-AAD Reagent (Merck, #4700-1510) was prepared by adding 2 µL of the reagent to 148 µL of 1× Assay Buffer BA, which was then added to the cell suspension and mixed thoroughly before analysing using a Guava^®^ Muse^®^ Cell Analyzer (Merck, #0500-3115).

### 4.3. Preparation of Protein Extracts and Immunoblotting

Whole-cell extracts (WCEs) prepared from cells at approximately 80% confluency were harvested by scraping in ice cold PBS solution, and cells were pelleted at 1000× *g* (5 min at 4 °C), then resuspended and lysed by 30 min incubation on ice in RIPA buffer (10 mM Tris-HCl pH 7.5, 0.5 mM EDTA, 150 mM NaCl, 0.1% SDS, 1% sodium deoxycholate and 1% Triton X-100) supplemented with a protease inhibitor cocktail [2 mM b-glycerophosphate, 1 µg/mL of 1:1:1:1 chymostatin: leupeptin: antipain: pepstatin A (CLAP), 100 µm of phenylmethylsulphonyl fluoride (PMSF), 20 mM Na_3_VO_3_, 2 mM MgCl_2_ and 4 U/mL of DNase (Quanta Biosciences, Beverly, MA, USA)]. Then, they were sonicated at 50 Hz (10 s) and 25 Hz (10 s) on ice. Protein concentration was determined via a Bradford assay. WCEs intended for pull-down assays were stored at −20 °C, while lysates for SDS-PAGE were boiled with loading buffer/SDS sample buffer [100 mM b-mercaptoethanol, 50 mM Trisma pH 6.8 with HCl, 10% glycerol (*w*/*v*), 2% SDS (*w*/*v*), 0.01% bromophenol blue (*w*/*v*)] at 95 °C for 5 min.

Protein samples were run on 10, 12 or 15% polyacrylamide gels at 200 V for 1 h in 1× running buffer (25 mM Tris,190 nM glycine, 1% SDS), then transferred onto 0.22 µM nitrocellulose membranes (Cytivia, Wilmington, DE, USA) in 1× transfer buffer (25 mM Tris, 190 nM glycine, 0.1% SDS 10% methanol) at 350 mA for 1.5 h. The membranes were blocked in blocking buffer [5% Marvel milk or 5% BSA in TBST (50 mM Tris-HC, 150 mM NaCl, 0.1% Tween 20, pH 8.0)] for 1 h at RT, then incubated overnight at 4 °C with primary antibodies as follows: anti-beta-actin (Proteintech, Manchester, UK, #66009 1/750); pan-VDAC1/2/3 (Cell Signalling Technologies (CST), Danvers, MA, USA, #D73D12, 1/1000); VDAC1 (Proteintech 55259, 1/1000), VDAC2 (Proteintech #11663, 1/1000), VDAC2 (AbCam, Cambridge, UK, #B37985, 1/1000), (VDAC3, Proteintech, #55260, 1/1000), SEH1 (AbCam #Ab218531, 1/1000); BCL-XL (AbCam #32370, 1/1000), Bak (AbCam #32371. 1/1000), Bax (AbCam #Ab32503, 1/1000), GFP (home-made, 1/300), GST (Cytiva, RPN1236V, 1/10,000). After washing the membranes 3 times in TBST, they were incubated with HRP-conjugated secondary antibodies for 1 h at RT: HRP-conjugated anti-rabbit (Dako, Santa Clara, CA, USA, #P0217; 1/1500); HRP-conjugated anti-mouse (Dako, #P0260; 1/1500); HRP-conjugated anti-goat (Dako, #P0449; 1/1000). After 2 washes with TBS, the blots were processed using the enhanced chemiluminescence (ECL) Western blot detecting reagent, which was used according to the manufacturer’s instructions (Cytivia RPN2106), then exposed to X-ray film (Cytivia, 28906837) or imaged with a ChemiDocMP Imaging System (Bio-Rad, Hercules, CA, USA). Band intensities of immunoblots were measured using Fiji (ImageJ 2.16) software.

### 4.4. GFP Trap

WCEs prepared from exponentially growing HeLa cells stably expressing GFP or survivin-GFP (approximately 300 µg of protein in 0.5 mL) were incubated with GFP-Trap^®^ A (ChromoTek, Martinsried, Germany, #208032-01) beads (4 °C, 2 h) with rotation. Next, the beads were pelleted by centrifugation (2500× *g*, 4 °C, 2 min) and washed twice in dilution buffer (150 mM NaCl; 10 mM Tris-HCl; 0.5 mM EDTA, pH 7.5). Finally, the beads were boiled (90 °C, 5 min) in 100 µL of 2× SDS sample buffer to release the bound proteins.

### 4.5. Co-Immunoprecipitation (Co-IP)

Protein A/G Agarose beads (#20421 Pierce) were washed once with sterile H_2_O and twice with cold PBS. WCEs were pre-cleared by 1 h incubation with the washed beads, followed by centrifugation for 300× *g* at 4 °C for 5 min. The cleared lysates (150 µg in 200 µL) were then incubated overnight with 1 µg of the protein-specific antibody or rabbit/mouse IgG at 4 °C on a rotor. The following day, the beads were washed again with PBS and incubated with the overnight lysate (immunocomplex) for 2 h (RT) on a shaker. The beads were washed three further times with cold PBS and pelleted via centrifugation at 300× *g* at 4 °C for 3 min. Proteins were subsequently eluted using 2 × SDS sample buffer at 95 °C for 5 min. Antibodies (1 μg per 150 μg WCE) used for IP were anti-Bak (AbCam #32371), Bax (AbCam #Ab32503), BCL-XL (Proteintech #10783-1-AP), survivin (CST #71G4B7), and pan-VDAC1/2/3 (CST; #D73D12).

### 4.6. GST Pull Down

GST and GST-tagged survivin (GST-SVN) were prepared as described in [23]. Briefly, 500 μg WCEs were precleared with 100 µL of 50% Glutathione Sepharose 4B bead slurry (4 °C, 1 h) with rotation. After that, the beads were pelleted and removed by centrifugation (500× *g*, 4 °C, 5 min). The WCEs were then incubated (4 °C, 2 h) with 100 µL of 50% Glutathione Sepharose 4B bead slurry to which recombinant GST or GST-SVN had been conjugated (see [23]). The beads were re-pelleted via centrifugation (500× *g*, 4 °C, 5 min) and washed in 500 µL of RIPA wash buffer 2 (150 mM NaCl, 50 mM Tris-HCl, pH8 and 1.5% NP-40) and RIPA wash buffer 3 (1.5% NP-40 and 50 mM Tris-HCl, pH8) for 20 min at 4 °C with rotation. After each wash, the beads were centrifuged (500× *g*, 4 °C, 5 min) and the supernatants were discarded. The beads were then boiled (90 °C, 5 min) in 100 µL of 2× SDS loading buffer. GST pull-down was also used for the initial Mass Spectrometry screen.

### 4.7. In Vitro Translation (IVT)

Proteins were translated in vitro using the TNT-T7-coupled reticulocyte Kit (Promega, Southampton, UK, L4610) in accordance with the manufacturer’s guidelines. An aliquot of the in vitro translated (IVT) protein was retained as “input”, and the remainder was incubated with GST beads for GST pull-down assays (see [23]). The GST beads were subsequently washed with TBST and TBS, and the pull-down products were eluted with 2× SDS sample buffer at 95 °C for 5 min. Plasmids used were pcDNA3.1 SEH1 (positive control, a gift from Dr. M Platani and Prof. WC Earnshaw, Edinburgh University, UK [24]; pcDNA 3.1 VDAC1, 2 and 3, a gift from Prof. J. Holthuis, Osnabruck University, Germany [20]).

### 4.8. Statistical Analysis

All statistical analyses were conducted using GraphPad Prism v10 Software.

## 5. Conclusions

To conclude, we have reported a novel interaction between survivin and the OMM porin, VDAC2, that is indirect and potentially mediated by pro- and anti-apoptotic Bcl2-family members, Bak, Bax and Bcl-XL. If this proposed mechanism is validated, it may open an exciting new avenue for exploring the anti-apoptotic properties of survivin, one that is distinct from other IAP family members. This discovery may be advantageous in cancer intervention, as many onco-therapies are designed to induce mitochondrial-mediated cell death, and these same therapies are antagonised by high expression of survivin that is apparent in all cancers.

## Figures and Tables

**Table 1:** VDAC proteins identified in MS screen.

Protein	Accession	GST-Control	GST-Survivin
VDAC1	P21796	2	**1**
VDAC2	P45880	4	8
VDAC3	Q9Y277	2	2

Numerical values indicate Total Spectrum Count (TSC) values (Scaffold) in the respective samples. Proteins were pulled down from asynchronous HeLa cell extracts using GST or GST-survivin. N = 1. Only VDAC2 gave a strong positive interaction with GST-Survivin compared to control (highlighted in grey).

## Data Availability

The original contributions presented in this study are included in the article/Supplementary Materials. Further inquiries can be directed to the corresponding author.

## Supplementary Materials

The following supporting information can be downloaded at: https://www.mdpi.com/article/10.3390/ijms27135707/s1.
