# Supplementary material for: Survivin Associates with VDAC2 and Bcl2-Family Proteins at the Mitochondrial Outer Membrane
Source: Int J Mol Sci. 2026 Jun 24;27(13):5707. doi: 10.3390/ijms27135707 (PMC13362165; doi:10.3390/ijms27135707)
Supplement: Supplementary file 1 [file ijms-27-05707-s001.zip › ijms-4300346-supplementary.pdf]

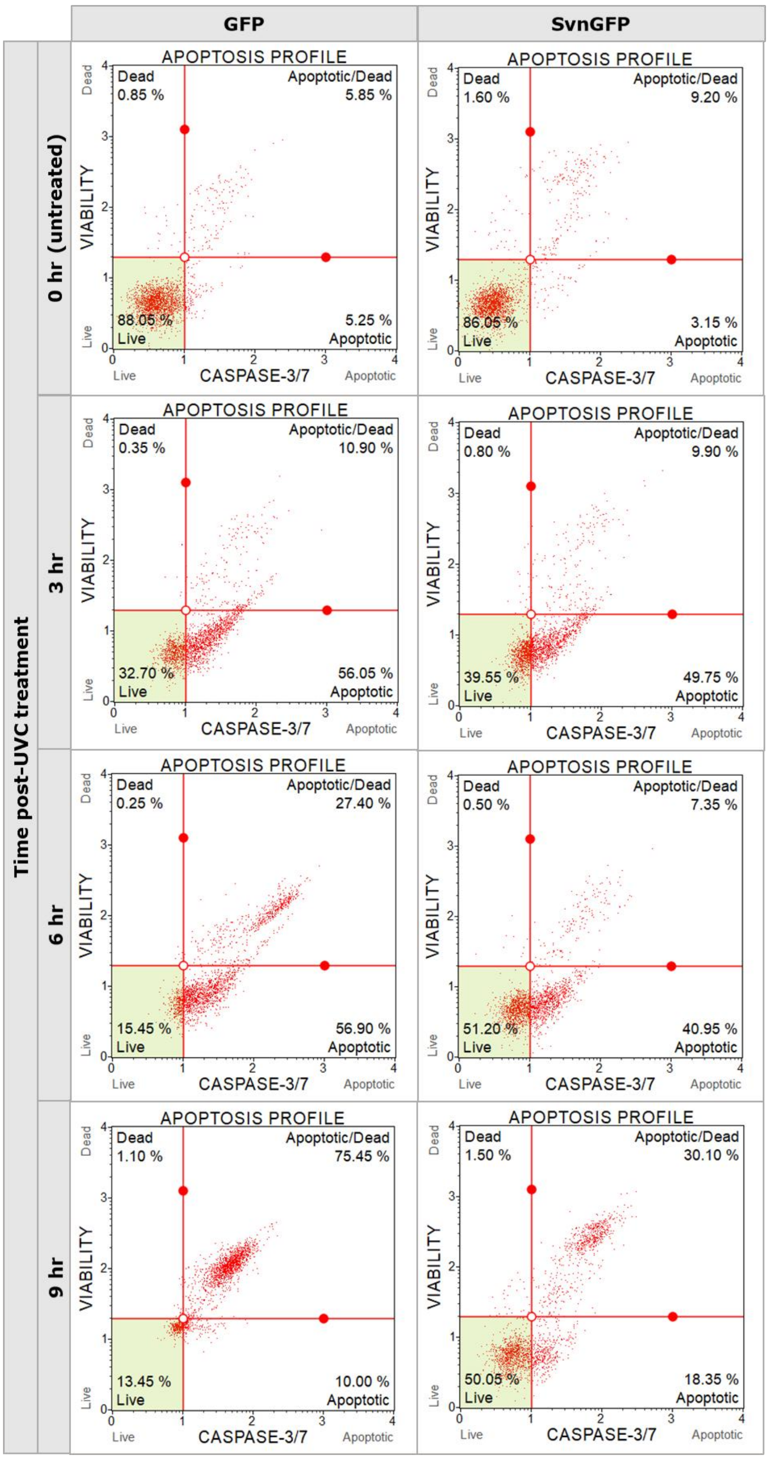

Figure S1: **Apoptotic status of HeLa cells following UVC treatment.** HeLa cells expressing GFP or SVN-GFP were exposed to 180 mJ·cm^2 UVC (254 nm) radiation and harvested every 3 h for 9 h and analysed by FACS. Gates were set based on 0 h (untreated) GFP controls to separate four distinct categories: live, early apoptotic, late apoptotic/dead and dead.

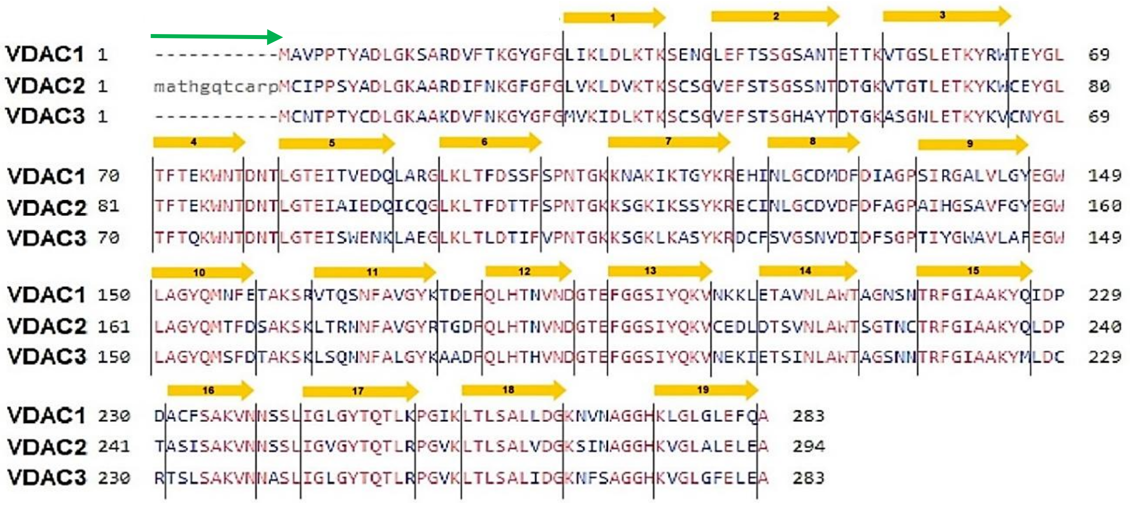

Figure S2 Multiple sequence alignment of human VDAC1, 2 and 3 protein sequences. Highly conserved amino acids are in red. The unique N-terminal 11 amino acids of VDAC2 are presented in lower case (black) and indicated with a green arrow. β-strands are indicated by yellow arrows. Created in COBALT using FASTA sequences from UniProt.

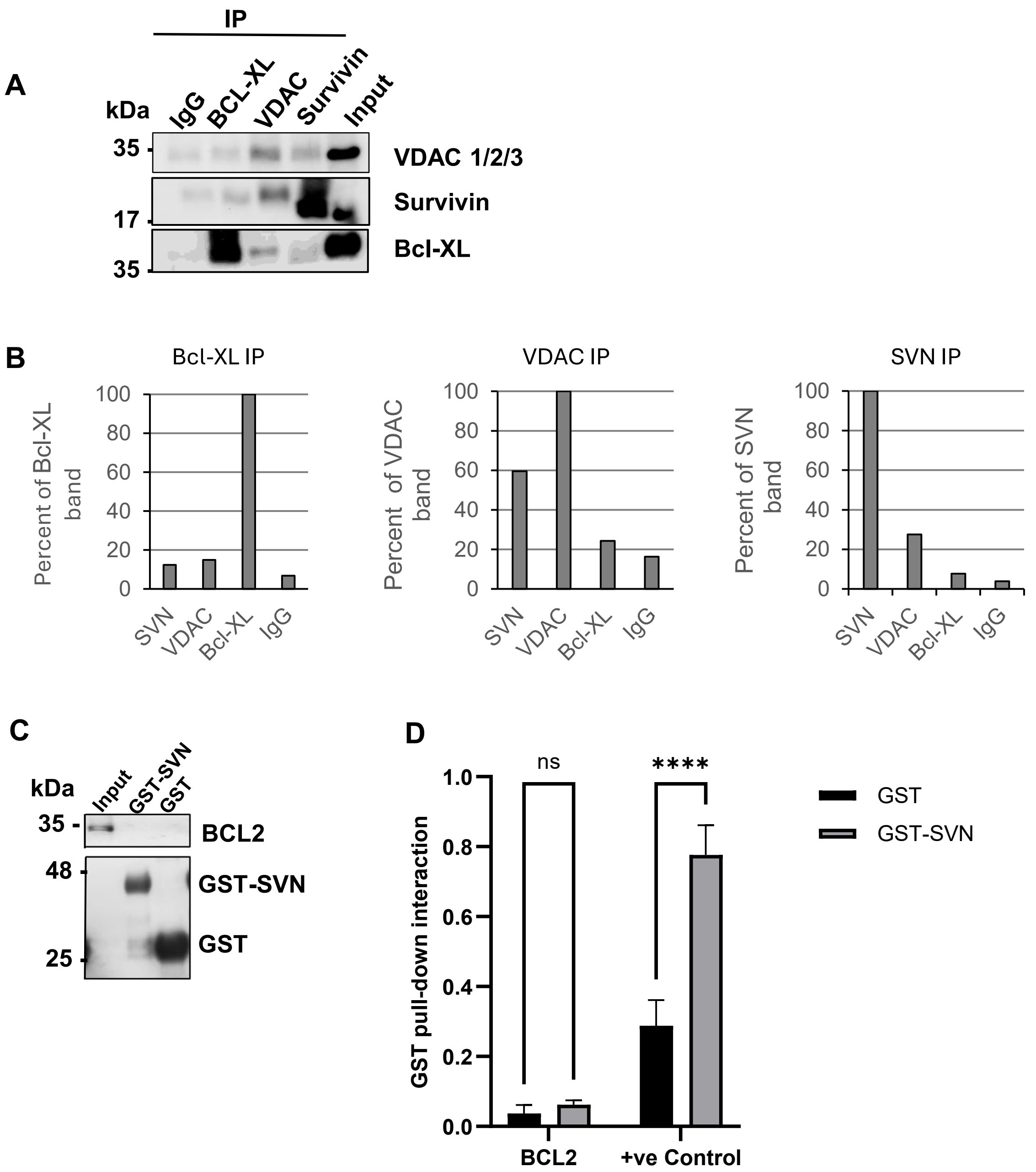

Figure S3: Co-IP of survivin, VDAC and Bcl-XL. (A) Immunoblot analysis of reciprocal co-immunoprecipitation of survivin, pan VDAC 1/2/3 and Bcl-XL from asynchronous HeLa cell extracts, see also Figure 4. N=2. **(B)** Quantification of each co-IP band relative to the band of the protein IP'd, which was set at 100%. **(C&D)** A GST pull down using asynchronous U2OS cells was interrogated with anti-Bcl2 antibodies. No interaction was seen whereas quantitation gave a significant interaction under the same conditions for the positive control. (N=3. Data are mean ± SD, student unpaired two-tailed t-test (****$p<0.0001$, ns not significant).
